# Supplementary material for: Risk of COVID-19 transmission on long-haul flights: During the COVID-19 pandemic
Source: PLoS One. 2024 Aug 16;19(8):e0309044. doi: 10.1371/journal.pone.0309044 (PMC11329108; doi:10.1371/journal.pone.0309044)
Supplement: Attachment — Submitted filename: Response to Reviewers.docx [file pone.0309044.s002.docx]

Dear editor and reviewers

We sincerely thank you for pointing out the improvement of the thesis carefully.

We did our best to fix it. Thank you a lot.

| Editor | Answer and correction |
| --- | --- |
| 1. Please ensure that your manuscript meets PLOS ONE's style requirements, including those for file naming. The PLOS ONE style templates can be found at  https://journals.plos.org/plosone/s/file?id=wjVg/PLOSOne_formatting_sample_main_body.pdf and  https://journals.plos.org/plosone/s/file?id=ba62/PLOSOne_formatting_sample_title_authors_affiliations.pdf | It was modified to fit the PLOS ONE style template according to PLOS ONE's style requirements. |
| 2. Please ensure that you include a title page within your main document. We do appreciate that you have a title page document uploaded as a separate file, however, as per our author guidelines (http://journals.plos.org/plosone/s/submission-guidelines#loc-title-page) we do require this to be part of the manuscript file itself and not uploaded separately. Could you therefore please include the title page into the beginning of your manuscript file itself, listing all authors and affiliations. | The main document has been modified to include a title page. |
| 3. Please amend your list of authors on the manuscript to ensure that each author is linked to an affiliation. Authors’ affiliations should reflect the institution where the work was done (if authors moved subsequently, you can also list the new affiliation stating “current affiliation:….” as necessary). | The list of authors on the manuscript has been modified to indicate both the institution where the work was performed and their current affiliations. |
| 4. Please include your full ethics statement in the ‘Methods’ section of your manuscript file. In your statement, please include the full name of the IRB or ethics committee who approved or waived your study, as well as whether or not you obtained informed written or verbal consent. If consent was waived for your study, please include this information in your statement as well. | We included the full ethics statement in the ‘Methods’ section and stated whether we agreed or not. |
| 5. Please include a separate caption for each figure in your manuscript. | A separate caption has been included for each figure in the manuscript. |
| 6. Please include captions for your Supporting Information files at the end of your manuscript, and update any in-text citations to match accordingly. Please see our Supporting Information guidelines for more information: http://journals.plos.org/plosone/s/supporting-information. | We have included a caption for the Supporting Information file at the end of the manuscript and updated in-text citations accordingly. |
| 7. Please review your reference list to ensure that it is complete and correct. If you have cited papers that have been retracted, please include the rationale for doing so in the manuscript text, or remove these references and replace them with relevant current references. Any changes to the reference list should be mentioned in the rebuttal letter that accompanies your revised manuscript. If you need to cite a retracted article, indicate the article’s retracted status in the References list and also include a citation and full reference for the retraction notice. | The reference list was reviewed to ensure completeness and accuracy. |
| Reviewer #2 | Answer and correction |
| 1) Please refine the abstract a bit more. | The abstract has been further supplemented. |
| 2) There are some awkward English expressions that are common among researchers whose native language is not English. If there is a more refined and clear expression, please reflect it. | We had received an English proofreading certificate to ensure more clear expression. |
